# Reconceptualizing the Role of Passion within Physical Therapy Education

**DOI:** 10.1007/s40670-026-02854-w

**Published:** 2026-09-21

**Authors:** Joel Sattgast, Stacia Hall-Thompson

**Affiliations:** 1 Department of Physical Therapy, Eastern Washington University, Spokane, WA USA; 2 Physical Therapy Department, Methodist University, Fayetteville, NC USA

**Keywords:** Passion, Physical therapy education, Curriculum, Professional identity formation, Resilience

## Abstract

Students entering physical therapy education frequently cite passion as central to their professional aspirations, yet its role in professional identity formation and across learning domains remains underdeveloped and poorly understood. Drawing on the Dualistic Model of Passion and informed by existing competency frameworks, this commentary reconceptualizes passion as an educational construct intersecting cognitive, affective, and psychomotor domains. We argue that passion often resides within the hidden curriculum and should be intentionally integrated into curricular design, assessment, and faculty modeling. Supporting autonomous internalization of personal and professional motivations may strengthen professional identity, resilience, and sustained engagement within the physical therapy profession.

## Background & Purpose

Students entering physical therapy education (PTE) frequently describe their professional aspirations in affective terms, most commonly stating, “I’m passionate about helping others” or “I want to follow my passion” [1]. These expressions reflect more than general career enthusiasm; rather, they signal early professional motivations that may influence learning engagement, persistence in the face of challenge, and the early development of professional identity [2–4]. While passion is routinely mentioned in student narratives, its role within professional identity formation (PIF) and across learning domains in PTE remains conceptually underdeveloped and inconsistently addressed.

Although philosophers such as Descartes recognized passion as a powerful force that both motivates behavior and requires regulation [5], contemporary conceptualizations of passion are more directly informed by Vallerand’s Dualistic Model of Passion (DMP) [2, 3, 6]. Within the DMP, two forms of passion exist - harmonious passion (HP) and obsessive passion (OP). While HP develops through autonomous internalization and is associated with flexibility, identity integration, and well-being, OP reflects controlled internalization and is characterized by rigidity, internal pressure, and an “at all costs” orientation [2, 7]. This distinction is particularly relevant to PTE as it reframes passion not as an emotional state [8] or personality trait, but rather as a dynamic process shaped by the quality of internalization and expressed through sustained affective and cognitive engagement within learning and professional development.

**Fig. 1:**
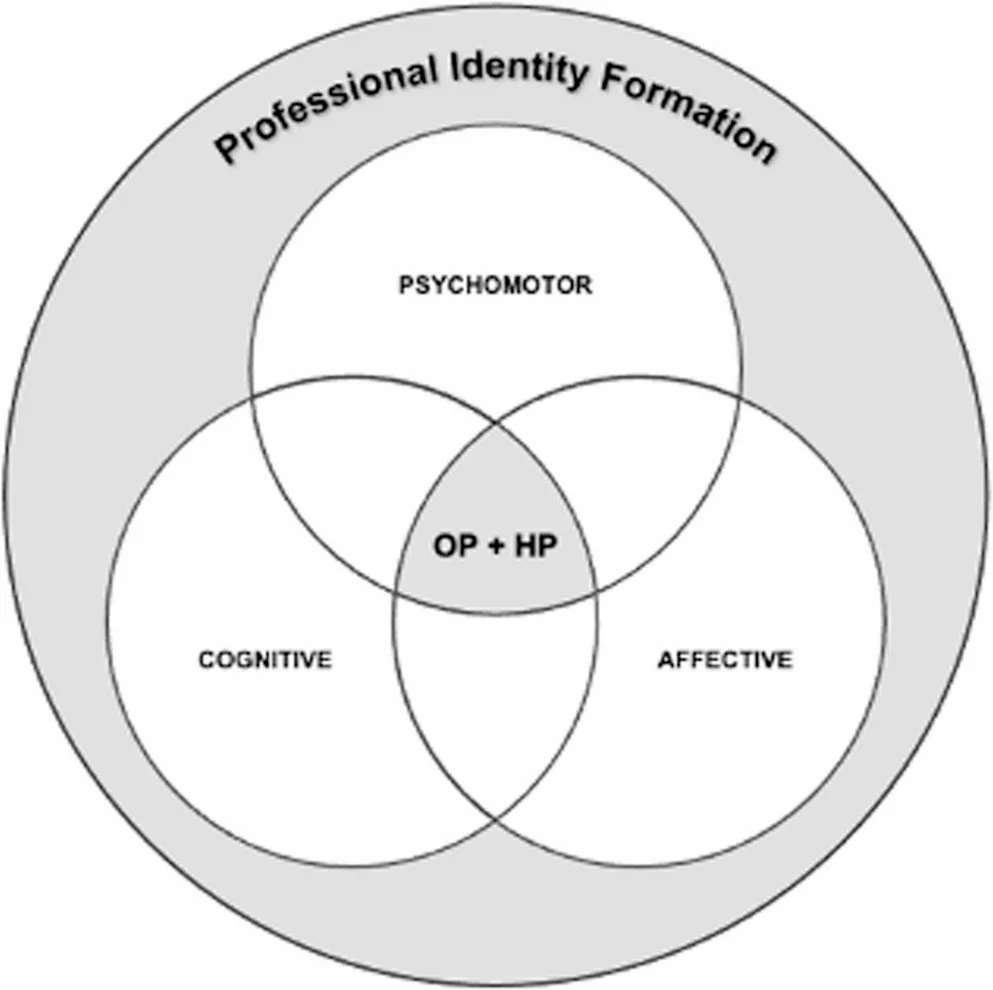
Passion (OP + HP) is an integrative and central construct within professional identity formation

The evolving integration of knowledge, skills, values, and the concept of self as a developing health professional underlies the PIF process and has been identified as a central goal of PTE [9]. Within this framework, PIF is supported through learning experiences that span the cognitive domain (knowledge acquisition and clinical reasoning), the psychomotor domain (skill development and performance), and the affective domain (values, attitudes, and professional behaviors) [9–11]. Passion may be conceptualized as a unifying construct that intersects all three domains (Fig. 1). Specifically, passion energizes cognitive engagement by sustaining attention and goal-directed learning, shapes affective development by reinforcing empathy and service to others and supports psychomotor learning by promoting persistence during repetitive and challenging skill acquisition [2, 3, 12].

Despite this integrative role, passion is rarely, if ever, explicitly acknowledged in PTE curricula. A recent PubMed search on 2/23/2026 using “passion” AND “physical therapy” AND “profession” yielded only 7 applicable results. Replacing “profession” with “curriculum” yielded only 1 appropriate result. While this preliminary search illustrates the scarcity of literature explicitly examining passion within PTE, this observation must not be interpreted as a formal systematic review. Rather, relevant scholarship may exist under related constructs such as PIF, vocation and/or calling, intrinsic motivation, and learner engagement among others.

Currently, existing competency frameworks emphasize critical thinking, communication, and professional behaviors [13], yet largely omit passion as a construct to be intentionally internalized, assessed, or guided. As a result, passion often resides within the hidden curriculum, modeled implicitly and discussed inconsistently, or within the null curriculum, where it is absent altogether. This omission risks underutilizing students’ intrinsic motivation, particularly amid increasing academic workload, financial pressure, and early-career burnout [14, 15].

Accordingly, the purpose of this commentary is twofold: (1) to reconceptualize passion as an educational construct relevant across the cognitive, affective, and psychomotor domains of physical therapy education, and (2) to propose educational considerations that support the autonomous internalization of students’ early professional motivations, thereby fostering HP that contributes to PIF and sustained professional engagement.

## Position & Rationale

Physical therapy education is currently undergoing substantial transition, with curricular redesigns emphasizing changes in credit load, competency-based outcomes, interprofessional collaboration, and preparation for evolving healthcare delivery models [16]. While these initiatives are necessary, they also risk narrowing educational priorities toward technical and cognitive competencies alone. Left unaddressed, this imbalance may further marginalize the affective domain and the motivational drivers within it, including passion, which plays a critical role in sustaining engagement and supporting PIF [17].

To mitigate this risk, we propose intentionally designing educational experiences that support the autonomous internalization of students’ professional motivations while integrating affective learning alongside cognitive and psychomotor development. Rather than functioning as an additional curricular outcome, passion and related motivations complement existing learning domains by shaping how students engage with knowledge acquisition, skill development, and professional values. Within the apprenticeships of professional education [11, 18], passion [5, 7] supports the development of:

1. Habits of the head *(cognitive domain)*: directing attention, sustaining effort during complex reasoning, and motivating intentional reflection.
2. Habits of the hand *(psychomotor domain)*: supporting the disciplined, repetitive practice required for clinical skill mastery.
3. Habits of the heart *(affective domain)*: reinforcing empathy, altruism, and resilience that ground professional behavior in meaning and purpose.

By situating passion within these “apprenticeships of professional education” [9, 10], educators are well positioned to support students’ emerging motivations and identity. Rather than remaining a generalized desire to “help others”, these motivations may become increasingly integrated within learners’ developing values, clinical experiences, and concept of their professional self. Within the DMP, the quality of this internalization influences whether passion is expressed in more harmonious or obsessive ways. HP becomes evident through flexible engagement with learning, persistence during psychomotor development, engaging in reflective practice, and ethical commitment; whereas, OP may emerge as rigidity, over-extension, and reduced flexibility under conditions of sustained performance pressure [7].

Leveraging existing competency frameworks and theory, passion also aligns well with transformative learning theory [19, 20], which emphasizes critical reflection and perspective shifts as mechanisms for reshaping ways of thinking (*head*), acting (*hand*), and feeling (*heart*). Transformative learning offers a process-oriented lens through which students may critically examine and reframe their initial expressions of passion, supporting the autonomous internalization of professional values and motivation. When educators design learning experiences that intentionally integrate these domains, passion is no longer an individual trait to be admired, but an educational energy to be guided and internalized.

Accordingly, PTE must explicitly engage passion as an educational resource through intentional curricular design and delivery [16, 20]. In doing so, challenges such as disengagement, identity fragmentation [15], and early-career distress may be attenuated, as students are equipped with the motivation, resilience, and self-awareness necessary for sustained professional formation [3, 21].

## Discussion & Conclusion

Recognizing passion as more than an initial emotional spark requires deliberate and systematic integration into the design and delivery of PTE. Despite students frequently entering PTE with strong professional aspirations and motivations, the education process by which these motivations become internalized, and ultimately influence PIF, resilience, and engagement, have received little attention. To advance the role of passion within internalization and PIF, we propose the following conceptual ‘next steps’ grounded in curricular theory and educational practice to illustrate how educational experiences may support the autonomous internalization of professional values and the development of HP. These considerations are summarized in Table 1.

**Table 1:** Operationalizing Passion within PTE - Conceptual ‘Next Steps’

Domain of Action	Conceptual Anchor	Curricular Implication	Practical Strategies	Educational Mechanism & Intended Outcome (DMP-Informed)
Curricular Positioning	Formal, actual, hidden, and null curricula	Move passion from hidden/null curriculum into explicit design	• Embed affective objectives across courses • Longitudinal threading of reflection • Align syllabi with affective competencies	Makes professional values and motivations explicit rather than relying on implicit learning alone, thereby supporting the autonomous internalization of professional values and fostering HP
Longitudinal Integration	PIF Carnegie’s habits of head, hand, heart	Integrate affective learning with cognitive and psychomotor development throughout the curriculum rather than in isolated learning experiences.	• Link clinical reasoning cases with value-based discussions • Integrate patient-centered ethics into skill labs • Reinforce reflective practice throughout clinical education.	Supports integration of knowledge, skills, and values, allowing PIF and HP to develop together through sustained educational experiences.
Pedagogical Design	Transformative learning theory Affective domain evaluation Experiential learning strategies	Provide structured opportunities to examine beliefs and motivations, through intentional educational experiences	• Guided narrative writing • Structured reflection and debriefing • Case-based reasoning discussions • Rubrics including reflection, resilience, and ethical engagement • Faculty guided dialogue	Encourages learners to examine, refine, and integrate their values through critical reflection, supporting autonomous internalization, identity integration, and the development of HP
Faculty Modeling	Hidden curriculum Role modeling Sustainable professionalism	Make sustainable professional commitment visible through explicit discussion of clinical practice, professional growth, and well-being	• Transparent dialogue about boundaries *(personal and professional)* • Modeling growth mindset • Explicit discussion of professional sustainability and well-being • Intentional mentorship and formative feedback	Demonstrate how meaningful professional engagement can be sustained across a career, helping learners distinguish HP from more rigid and controlled motivational patterns, while also reinforcing healthy professional identity development.
Scholarly Inquiry	Evidence-informed pedagogy ACAPT Excellence Framework	Develop a research agenda examining passion-informed educational practices and their relationship to professional development	• Longitudinal tracking of passion across the curriculum • Examine relationships among passion, burnout, resilience, PIF, and well-being • Evaluate the impact of affective integration strategies	Establish an empirical foundation for educational strategies that support autonomous internalization, HP, PIF, resilience, and sustained professional engagement.

Educational researchers have described four curricular spaces within professional education: the formal curriculum (explicit learning objectives and content), the actual curriculum (what is enacted in practice), the hidden curriculum (implicit values and norms), and the null curriculum (what is often omitted entirely) [12, 22]. Within PTE, passion and related affective domain behaviors most often reside within the hidden curriculum – implicitly modeled, inconsistently discussed, and rarely assessed. While implicit exposure may influence learners, it lacks the intentionality necessary to consistently support the autonomous internalization of professional values and may inadvertently reinforce more rigid and controlled forms of internalization, particularly in environments characterized by high workload and performance pressure (e.g., future clinical practice).

To address this imbalance, the affective domain, and passion as a motivational construct within it, must be brought out of the hidden and null curricula and into explicit curricular design [23]. This requires intentionally threading affective-domain learning throughout all aspects of the curriculum rather than relegating it to isolated professional seminars or orientation-based content. Consistent with prior work [17], affective development is most effective when embedded longitudinally and aligned with cognitive and psychomotor learning rather than treated as ancillary. Within this model, passion is positioned not as a fixed personality trait, but as a motivational process that can be intentionally supported through educational experiences as students develop further professional competence and identity integration [24].

Embedding passion across the curriculum necessitates learning experiences that help students translate abstract concepts into meaningful clinical action. Experiential learning strategies - including structured reflection [18, 25], guided narrative writing [25, 26], and case-based discussion [27] - provide opportunities for learners to critically examine their values, motivations, and professional experiences. Collectively, these experiences may serve to further support the autonomous internalization of professional values and PIF, thereby fostering HP. Importantly, such approaches are closely aligned and complement transformative learning theory [19, 28], which emphasizes critical reflection and perspective transformation as catalysts for professional growth.

Assessment practices must also be examined through this lens as they represent a critical yet underutilized opportunity to make passion educationally actionable rather than merely aspirational. Rather than attempting to assess passion directly, formative and summative assessment strateiges should focus on observable affective-domain behaviors associated with HP, including reflective capacity, ethical engagement, resilience, professional judgement, and professional identity development. Rubrics and feedback structures that explicitly acknowledge these behaviors not only reinforce their value but also help identify learners who may require additional guidance in navigating personal and professional challenges. Incorporating these considerations, assessment becomes educationally actionable by supporting the developmental processes associated with HP, while also avoiding the reduction of passion to an aspirational construct.

Faculty and clinical mentors also play a pivotal role in modeling behaviors and in shaping the educational environments in which students interpret and internalize sustainable expressions of passion across a professional lifespan [24]. Transparent discussions of professional challenges, boundary-setting, growth mindset, and work-life integration help learners observe how meaningful engagement can be sustained throughout a career rather than depleted early. Such modeling [24] reinforces the distinction between OP and HP, demonstrating that commitment to excellence does not require self-sacrifice or emotional overextension.

Finally, continued scholarly inquiry into passion-informed pedagogy within PTE is needed. Future research should examine how motivational internalization and passion evolve across stages of professional education, how these processes are influenced by curricular and clinical experiences, and which pedagogical strategies most effectively support HP. Longitudinal studies examining relationships among passion, PIF, resilience, and professional well-being would further strengthen the evidence base and inform best practices for curriculum design and faculty development.

For students entering PTE, strong professional aspirations, often interpreted as passion, and a desire to serve others remain central. Yet the educational processes through which these motivations become internalized, ultimately influencing PIF, resilience, and sustained professional engagement, have received comparatively little attention. In this commentary, we propose that passion is best recognized not as a fixed personal characteristic or transient emotional state, but as a motivational process which may be positively shaped through professional education. Drawing on the DMP, we argue that educators play an important role not in creating passion itself, but in meaningfully designing learning environments that support the autonomous internalization of professional values and identity.

Reconceptualizing passion in this manner shifts from inquiring whether students possess passion to instead considering how professional education may shape its development over time. By intentionally integrating affective-domain learning opportunities alongside cognitive and psychomotor development, physical therapist educators may better prepare clinicians who are not only technically competent, but also reflective, resilient and capable of sustaining meaningful professional engagement throughout their careers and within an evolving healthcare landscape.

## References

[1] USC Doctor of Physical Therapy Blog. Why become a physical therapist? USC Division of Biokinesiology and Physical Therapy Doctor of Physical Therapy Program. https://dpt.usc.edu/blog/why-become-a-physical-therapist/. Accessed July 17, 2026.

[2] Vallerand R, Koestner R, Mageau G, Gagne M, Ratelle C, Le’onard M, Marsolais J, Blanchard C. Les passions de L’ame: on obsessive and harmonious passion. J Personality Social Psyc. 2003. 10.1037/0022-3514.85.4.756. 14561128

[3] Jachimowicz J, Wihlerb A, Baileya E, Galinskya A. Why grit requires perseverance and passion to positively predict performance. PNAS. 2018. 10.1073/pnas.1803561115. PMC6176608 30224491

[4] Harman K, Sim M, LeBrun J, et al. Physiotherapy: an active, transformational, and authentic career choice. Physiother Theory Pract. 2021. 10.1080/09593985.2019.1639230. 31293200

[5] Vallerand RJ. The role of passion in sustainable psychological well-being. Psychol Well-Being: Theory Res Pract. 2012. 10.1186/2211-1522-2-1.

[6] Newport C. Follow a career passion? Let it follow you. *New York Times.* September 12, 2012. https://www.nytimes.com/2012/09/30/jobs/follow-a-career-passion-let-it-follow-you.html?smid=tw-share. Accessed Jul 17, 2026.

[7] Lafrenière MA, St-Louis AC, Vallerand RJ, Donahue EG. On the relation between performance and life satisfaction: The moderating role of passion. Self Identity. 2012. 10.1080/15298868.2011.616000.

[8] Page D. May. The spark of passion. Seeing parallels in a devastating event. *PT in MOTION*. 2017. 60–2.

[9] Gwyer J, Hack LM, Jensen GM, Mostrom E, Nordstrom T. Philosophy of excellence. In: American Council of Academic Physical Therapy. Blueprint for excellence in academic physical therapy. 2024. www.acapt.org

[10] Jensen GM, Paschal KA, Shepard KF. Curriculum design for physical therapy educational programs. In: Jensen GM, Mostrom E, editors. Handbook of teaching and learning for physical therapists. 3rd ed. St. Louis: Elsevier Butterworth-Heinemann; 2013. pp. 2–18.

[11] Jensen GM, Mostrum E, Hack LM, Nordstrom T, Gwyer J, Part I. Exploring Excellence in Professional Education. Educating Physical Therapists. New York: Routledge; 2024.

[12] Shepard KE, Jensen GM. Curriculum design for physical therapy education programs. In: Jensen GM, Mostrom E, editors. Handbook of teaching and learning for physical therapists. Woburn: Butterworth-Heinemann; 2002. pp. 1–33.

[13] Jette DU, Portney LG. Construct validation of a model for professional behavior in physical therapist students. Phys Ther. 2003;83(5):432–43. 12718709

[14] Jette A. Addressing Burnout in the Physical Therapy Workforce. Phys Ther. 2023. 10.1093/ptj/pzad150. https://doi.org/. 37930245

[15] Smith A, Ellison J, Bogardus J, Gleeson P. Development of burnout in physical therapist students and associated factors: a study during COVID-19. J Phys Ther Edu. 2022. 10.1097/JTE.0000000000000239.

[16] Blackinton MT, Nixon-Cave K, Robertson E, Tilson J. Curricular design and delivery in contemporary doctor of physical therapy education. In: American Council of Academic Physical Therapy. Blueprint for excellence in academic physical therapy. 2024. www.acapt.org

[17] Berg-Carramusa CA, Mucha MD, Somers K, Piemonte N. The time is now: Leveraging the affective domain in pt education and clinical practice. J Phys Ther Edu. 2023. 10.1097/JTE.0000000000000271. 38478823

[18] Jensen GM, Learning. What matters most. Phys Ther. 2011. 10.2522/ptj.2011.mcmillan.lecture. 21903733

[19] Mezirow J. An overview of transformative learning. In: Sutherland P, Crowther J, editors. Lifelong learning: concepts and contexts. New York: Routledge; 2007. pp. 24–38.

[20] Burns SA, Gilliland SJ, Giuffre SM, Muñecas T. Student success and learning. In: American Council of Academic Physical Therapy. Blueprint for excellence in academic physical therapy. 2024. www.acapt.org

[21] Stillman J. New Harvard research: to be successful, chase your purpose, not your passion. New studies show purpose gives you resilience, and that’s what will get you ahead. *Inc.* November 18, 2019. https://www.inc.com/jessica-stillman/new-harvard-research-to-be-successful-chase-your-purpose-not-your-passion.html. Accessed July 17, 2026.

[22] Hafferty FW, O’Donnell JF. The hidden curriculum in health professional education. Hanover: Dartmouth College; 2015.

[23] Dutton LL, Sellheim DO. The informal and hidden curriculum in physical therapist education. J Phys Ther Edu. 2014. 10.1097/00001416-201407000-00008.

[24] Plack MM, Healey WE, Huhn K, Costello E, Maring J, Hilliard MJ. Navigating student challenges: from the lens of first-year doctor of physical therapy students. J Phys Ther Edu. 2022. 10.1097/JTE.0000000000000212.

[25] Paul RW. The logic of creative and critical thinking. Am Behav Sci. 1993. 10.1177/0002764293037001004.

[26] Paul RW. Dialogical thinking: critical thought essential to the acquisition of rational knowledge and passions. In: JB Baron & RJ Sternberg, editors. Teaching thinking skills: theory and practice. WH Freeman/Times Books/Henry Holt & Co: 1987. pp. 127–148.

[27] ten Cate O, Custers EJ, Durning SJ. Principles and practice of case-based clinical reasoning education: a method for preclinical students. Springer; 2018. 31314234

[28] Kitchenham A. The evolution of John Mezirow’s transformative learning theory. J Trans Educ. 2008. 10.1177/1541344608322678.

